# Modified Renshen Wumei Decoction Improves Qi-Yin Deficiency Diarrhea by Regulating the Gut Microbiotas and Metabolites in Rats

**DOI:** 10.4014/jmb.2412.12037

**Published:** 2025-06-23

**Authors:** Junqi Zhao, Zhiwei Guan, Shuhua Fan, Jianli Qiu, Yan Xu, Qiong Zhao

**Affiliations:** 1 Pediatric Hospital, First Affiliated Hospital, Henan University of Chinese Medicine, Zhengzhou 450000, P.R. China; 2 School of Pediatrics, Henan University of Chinese Medicine, Zhengzhou 450046, P.R. China; 3 Chengdu University of Traditional Chinese Medicine, Chengdu 610075, P.R. China

**Keywords:** Modified Renshen Wumei Decoction, Qi-yin deficiency syndrome with diarrhea, 16S rRNA gene sequencing, metabolome sequencing

## Abstract

The hub metabolites and hub microorganisms that play an important role in the intervention of Qi-yin deficiency syndrome with diarrhea by Modified Renshen Wumei Decoction (MRWD) are still unclear. Therefore, we explored it based on multi-omics analysis. A total of 32 Sprague-Dawley rats were collected and randomly allocated into different groups. Subsequently, blood samples and fecal samples were collected from all 32 rats for non-targeted metabolome sequencing and 16S rRNA gene sequencing respectively. Among them, fecal samples of 24 rats were also used for targeted metabolomics sequencing (6 rats in each group). Pathological findings, and D-lactate, diamine oxidase (DAO), aquaporin 3 (AQP3), and aquaporin 8 (AQP8) levels in serum and colon samples were evaluated after the 21-day trial, and the results showed that diarrhea caused intestinal injury, which was ameliorated by infusion of MRWD. Based on multi-omics sequencing analysis, five microorganisms were superior to the positive control in MRWD intervention. Moreover, *Eisenbergiella*, *Corynebacterium*, and *unidentified Oscillospiraceae* exhibited significant discriminatory capabilities between groups C and D, groups B and C, as well as groups A and C; thus they were identified as hub microorganisms. On the other hand, significant differences in metabolites were observed among different groups with respect to the metabolome. These metabolites exhibited significant enrichment in metabolic pathways such as Butanoate metabolism, Propanoate metabolism, and Pyruvate metabolism. Further correlation analysis revealed that 2-Methylbutyrate was identified as a hub metabolites associated with blood and fecal microorganisms. Moreover, there were complex regulatory relationships between these hub microbes and hub metabolites.

## Introduction

Diarrhea is defined as an increase in the number of bowel movements and/or a change in stool character, manifested by thin or watery stools, and is particularly common in children; it is also one of the leading causes of high morbidity and mortality in children under the age of five years [1, 2], and although mortality rates associated with acute diarrhea are declining, prolonged (7-13 days) and persistent (duration ≥14 days) diarrhea remains a great challenge [3, 4].

From the perspective of Chinese medicine, prolonged diarrhea is mostly characterized by prolonged diarrhea or diarrhea that does not resolve, which ultimately leads to a deficiency of both Qi-yin, and is often treated with Modified Renshen Wumei Decoction (MRWD), which has a good clinical efficacy. Traditional Chinese medicine has significant efficacy in the treatment of diarrhea. Traditional Chinese medicine contains polysaccharides, peptides, glycoproteins, lipids and their metabolic derivatives, etc., which can have an impact on the abundance and diversity of microorganisms [5, 6], and the intestinal flora regulates a variety of energy metabolisms, which include the production of short-chain fatty acids, which play an important role in regulating intestinal cellular function, and can protect the host organism from the damage caused by metabolic disorders, and may also be associated with inflammatory reactions and other types of intestinal diseases, such as diarrhea. They can protect the host organism from metabolic disorders and may be associated with inflammatory responses and other intestinal diseases [7], therefore, the intestinal flora can produce a large amount of short-chain fatty acids to maintain the homeostasis of the intestinal immune system.

Currently, the use of gut flora and its metabolites to understand the relationship between diseases and microorganisms has been widely used. However, little is known about the interactions between gut microbes and metabolites and how they affect the development of Qi-yin deficiency syndrome with diarrhea. Therefore, in this study, metabolome sequencing combined with microbiome sequencing was used to jointly analyse the pivotal metabolites and pivotal microorganisms that play important roles in the intervention of MRWD in rats with Qi-yin deficiency syndrome with diarrhea, as well as their potential mechanisms of action in diarrhea, which will provide new directions for the treatment of diarrhea.

## Materials and Methods

### Preparation of Decoction

**Preparation of 30% senna leaves.** The senna (300 g) was immersed in pure water (1,000 ml) and soaked for 5 min after thorough stirring. Subsequently, the solution was boiled with a large fire and then decocted with a small fire for 15 min to avoid evaporation. After that, the solution underwent filtration using a four-layered 200-mesh filter cloth to eliminate impurities and excess water from the senna leaves by means of thorough squeezing. The filtrate was subsequently collected and subjected to concentration using a rotary evaporator under reduced pressure. This was followed by dissolution and adjustment of the volume to 1,000 ml. After filtration and sterilization, the 30% senna leaves was labeled and stored in a refrigerator at 4°C.

**Preparation of MRWD.** MRWD consists of *Renshen Radix et Rhizoma* (8 g), *Mume Fructus* (8 g), *Crataegi Fructus* (5 g), *Dioscoreae Rhizoma* (5 g), *Pogostemon cablin* (5 g), *Poria* (5 g), *Nelumbinis Semen* (5 g), *Zingiberis Rhizoma* (1 g), and *Glycyrrhizae Radix et Rhizoma* (3 g) with a total weight of 50 g. Initially, 500 ml of pure water was added to the mixture, which was then boiled for one hour and filtered using a 200-mesh filter. Next, an additional 400 ml of distilled water was added to the mixture, boiled for one hour, and filtered using a 200-mesh filter. Afterwards, the filtrates from both steps were combined and concentrated under reduced pressure to obtain a concentration of 1.74 g/ml. The MRWD was sterilized and stored in a refrigerator at 4°C.

### Subjects and Sample Collection

In this study, Sprague-Dawley (SD) male rats aged 3-4 weeks and weighing 60-70 g (provided by Henan Skobes Biotechnology Co., Ltd., China) were utilized as experimental animal models for multi-omics analysis. All animals were raised in a specific pathogen-free (SPF) environment with a lighting cycle of 12 h/12 h, indoor temperature controlled at around 25°C, and good ventilation. The experimental design and acquisition of animals adhered to ethical guidelines and received approval from the Experimental Animal Welfare Ethics Committee of Henan University of Traditional Chinese Medicine (ethics number: IACUC-202308037).

### Establishment of Diarrhea Model Rats

After 7 days of adaptive feeding, 32 experimental rats were randomly divided into blank group, model group, treatment group, and positive control group according to random number table, with 8 rats in each group. With the exception of the blank group, the experimental rats in all other groups were administered 30% senna leaves (20 ml/kg) via gavage. After 1 h of gavage, the rats were placed in water with a water depth of 40 cm and a temperature of 30°C for weight-bearing swimming (the tail root of the rat was wrapped in a fuse with a weight of 5% of the rat’s body weight), with exhaustion as the degree (the rat’s nasal tip entered the water surface for 5s). Subsequently, the rats were gently dried with a towel to eliminate fur moisture, and the diarrhea model was induced through intermittent fasting (no diet but drinking water on the following day) for a total duration of 14 days. After the modeling process, the model underwent an evaluation based on various parameters including body weight, hair condition, and mental state to determine the success of establishing the diarrhea model. After the successful establishment of the diarrhea model, the treatment group received MRWD at a dosage of 20 ml/kg via gavage, while the positive control group was administered live combined *Bacillus subtilis* and *Enterococcus faecium* at a dosage of 20 ml/kg. Additionally, both the blank group and model group were given an equal volume of normal saline via gavage. The rats in the 4 experimental groups were administered once daily for 7 consecutive days. After 7 days of treatment, the rats were forbidden to eat but could drink water for 12 h. Subsequently, the rats were anesthetized via intraperitoneal injection of pentobarbital sodium (2.5 ml/kg), and approximately 4-6 ml of blood was collected from the abdominal aorta for non-targeted metabolomics sequencing. Following this, fecal samples of rats were collected for 16S rRNA gene sequencing and metabolome sequencing. Importantly, the difference was that the samples used for targeted metabolomics sequencing were 24 fecal samples.[Table T1]

### Enzyme-Linked Immunosorbent Assay (ELISA)

D-lactate,DAO determination using enzyme-linked immunosorbent assay test kit, consistent with the manufacturer's instructions (Wuhan Cloud-Clone Crop Technology, China).

### Western Blot Analysis

Rat colon tissue was collected, lysate and protease inhibitor were added, centrifuged at 12,000 rpm for 15 min, and the protein concentration was determined by BCA method, the protein samples were separated by 10% SDS-PAGE gel electrophoresis, and the samples were transmuted semi-dry, closed incubated with skimmed milk for 2 h. The samples were added with AQP3, AQP8 and the internal reference GAPDH at 4°C overnight, and then the samples were incubated with the secondary antibody at 37°C for 2 h on the following day and finally add ECL colour developer dropwise for fixation.

### 16S rRNA Gene Sequencing and Data Preprocessing

Fecal samples were collected from each group, and total genomic DNA was extracted. The concentration and purity of the extracted DNA were assessed by electrophoresis on a 1% agarose gel. Subsequently, the DNA was diluted to a final concentration of 1 ng/μl using sterile water. Primers targeting the variable V4 region of the 16S rRNA gene were designed for amplification. For PCR amplification, all reaction mixtures contained 15 μl Phusion High-Fidelity PCR Master Mix (New England Biolabs, China), 0.2 μM primers, and 10 ng genomic DNA template. The initial denaturation step was carried out at 98°C for 1 min, followed by a cycling program consisting of 30 cycles at 98°C (10s), 50°C (30s), and 72°C (30s). A final extension step was performed at 72°C for 5 min. The resulting PCR products were purified using magnetic bead purification technology. After that, a library was constructed, and its quantification was determined using Qubit and Q-PCR. After quality assessment, the constructed library underwent PE250 computer sequencing on NovaSeq6000. Additionally, we employed splicing and filtering techniques on the raw data to acquire high-quality Tags data, thereby ensuring the precision of our analysis.

### Metabolomics Sequencing and Data Preprocessing

The non-targeted metabolome data were analyzed based on 32 blood samples that were collected. Initially, the collected blood samples were thawed on ice and then metabolites were extracted using pre-cooled 50% methanol. After vortex mixing, the mixture was incubated at room temperature and subsequently stored at a low temperature. To separate the supernatant, the extraction solution underwent centrifugation and was transferred to a new 96-well plate for cryogenic storage in preparation for LC-MS analysis. Prior to analysis, each extract was added to a prepared QC sample. For chromatographic separation, an ultra-high performance liquid chromatography coupled with tandem mass spectrometry (UHPLC-MS/MS) system (Vanquish Flex UHPLC-TSQ Altis, Thrmo Scientific Corp., Germany) was employed utilizing a specific column Waters ACQUITY UPLC BEH C18 (1.7 μm, 2.1 × 100 mm) under gradient elution conditions. The mass spectrometer operated in negative multiple reaction mode (MRM). Throughout the acquisition process, quality control samples were collected to evaluate the stability of the mass spectrometer's performance. Additionally, targeted metabolomics sequencing was conducted to analyze short-chain fatty acids in 24 fecal samples.

### Analysis of Dilution Curve, Species Accumulation Curve and Rank Abundance Curve

In order to assess the rationality of sample sequencing and depth, as well as the richness and evenness of samples, dilution curves, species accumulation curves, and hierarchical abundance curves were generated. In the dilution curve, the x-axis represents the amount of sequencing data, while the y-axis represents the corresponding alpha diversity index. When the curve reaches a plateau, it indicates that an optimal amount of sequencing data has been obtained, and further increase in data will not significantly affect the alpha diversity index. Alpha diversity primarily captures richness and evenness using metrics such as Chao1, Observed species, Shannon's index, and Simpson's index. The species accumulation curve plots the sample size on the abscissa and the number of feature sequences after sampling on the ordinate. It provides an overall measure of the rate at which new feature sequences are encountered through continuous sampling. Consequently, it serves as a valuable tool for assessing sample adequacy. A steep incline in the curve suggests insufficient sampling, necessitating an increase in sample size. Conversely, a plateau indicates sufficient sampling has been achieved, enabling data analysis to proceed. The rank abundance curve depicts the sequence number of features sorted by their abundance on the horizontal axis, while the vertical axis represents the relative abundance of corresponding feature sequences. Each sample is distinguished by a distinct colored line. The width of the curve reflects species richness along the horizontal direction, with greater span indicating higher species richness on the x-axis. Smoothness of the curve in the vertical direction indicates uniformity of species within a sample, where a smoother curve signifies more evenly distributed species.

### Microbial Diversity Analysis and Relative Abundance Analysis

According to the Alpha diversity analysis results of Chao1, Shannon's index, observed species and Simpson's index, a Wilcoxon test was conducted to analyze the differences between two groups of the MRWD group (A), positive control group (B), model group (C), and blank group (D). Violin plots were generated to visually represent these differences. Subsequently, microbial Beta diversity was assessed through Principal Coordinate Analysis (PCoA) and Non-metric Multidimensional Scaling (NMDS) analyses. Specifically, the vegan package was employed to perform PCoA analysis on groups A, B, C, and D in 16s sequencing data using four algorithms - Bray-Curtis, Jaccard, Weighted Unifrac, and Unweighted Unifrac distances. Unlike PCoA which relies on distance matrix values for dimensionality reduction calculation, NMDS determines dimensionality reduction based on distance ranking order. The reliability of these algorithms' results is indicated when the stress values of NMDS for all four algorithms were ≤ 0.2. Furthermore, in order to assess the specific alterations in the intestinal microbiota, the species abundance table at both phylum and genus levels was generated based on microbial sequencing data and annotation results from the sample species abundance table. Additionally, the species composition of different groups was analyzed using this abundance table, and a bar chart depicting relative microbial abundance of the top 10 microbial phyla and top 20 microbial genera was created using the ggplot2 package (v 3.4.4) [8].

### Identification of Candidate Microorganisms

In order to identify the differential microorganisms between the model group and the blank group (C vs D), the positive control group and the model group (B vs C) and the MRWD group and the model group (A vs C), Linear discriminant analysis Effect Size (LEfSe) analysis was conducted at the genus level. The resulting differentially abundant taxa were designated as DETs1, DETs2, and DETs3 for comparisons of C vs D, B vs C, and A vs C, respectively. The threshold was set to LDA score > 4 and *p* < 0.05.

In order to identify the microorganisms associated with the intervention of live combined *B. subtilis* and *E. faecium*, we conducted a genus-level analysis. The ggvenn package (v 0.1.9) [9] was used to draw the Venn diagram to intersect the up-regulated microorganisms in DETs1 with the down-regulated microorganisms in DETs2, and the down-regulated microorganisms in DETs1 intersected with the up-regulated microorganisms in DETs2. Then, the two intersecting microorganisms were taken and combined to obtain the microorganisms with opposite expression trends between DETs1 and DETs2, which were recorded as opposites1. Similarly, microorganisms with opposite expression trends between DETs1 and DETs3 were also obtained, denoted as opposites2. These microorganism were related to the intervention of MRWD. In order to obtain the microorganisms related to the intervention of MRWD better than live combined *B. subtilis* and *E. faecium*, the opposite2 and opposite1 were subtracted, and the obtained microorganisms were named as candidate microorganisms.

### Recognition of Hub Microorganisms

In order to identify the key microorganisms that significantly contribute to the differentiation between groups in the A-C microbiome sequencing data, a random forest (RF) model was constructed using the caret package (v 6.0.94) [10] based on ASV characteristics of candidate microorganisms. Subsequently, the pROC package (v 1.18.0) [11] was utilized to generate the receiver operating characteristic (ROC) curve and calculate the area under the curve (AUC) value, which served as an indicator for evaluating model prediction accuracy. Microorganisms with AUC values exceeded 0.7 were defined as key microorganisms. Furthermore, we assessed the diagnostic capability of these key microorganisms in distinguishing between groups A vs C, B vs C, and C vs D. Notably, candidate microorganisms with AUC values exceeding 0.7 in all three group comparisons were designated as hub microorganisms.

### Principal Component Analysis (PCA)

In order to assess the extent of variation within and between sample groups, PCA was conducted on the processed metabolic data of all fecal and blood samples using the stats package. This analysis aimed to evaluate the overall distribution trend among different sample groups and identify any distinct clusters. Additionally, Orthogonal Partial Least Squares Discriminant Analysis (OPLS-DA) was performed between A and C, B and C, and C and D groups using the mixOmics package in both fecal and blood metabolome sequencing data. This purpose was to establish a relationship model between metabolite expression and sample categories, effectively separate samples, and predict sample categories by obtaining Variable Importance in Projection (VIP) values for each metabolite. Furthermore, the permutation test (*n* = 200) was employed to assess whether the OPLS-DA model suffered from overfitting.

### Identification of Hub Metabolites

In order to investigate the biological functions and signaling pathways associated with metabolites, MetaboAnalyst software was utilized to convert the compound names of differential metabolites into Kyoto Encyclopedia of Genes and Genomes (KEGG) IDs. Subsequently, employing the KEGG database as a reference, Metaboanalyst software was employed for analyzing the metabolic pathways of these metabolites (*p* < 0.05). In order to identify hub metabolites, Spearman correlation analysis was performed on the hub microorganisms and metabolites using the psych package (v 2.1.6) [12] in all samples of both metabolome sequencing data and microbiome sequencing data. Hub metabolites were defined as those strongly associated with the hub microorganisms in both blood and fecal samples.

### Construction of Hub Metabolite-Hub Microorganism Regulatory Network

In order to investigate the relationship between hub metabolites and hub microorganisms, Spearman analysis was performed using the psych package (|cor|>0.3, *p* < 0.05). Additionally, Cytoscape software (v 3.10.1) [13] was utilized to construct a network diagram illustrating the regulatory network of hub microorganisms and hub metabolites.

### Statistical Analysis

Based on R software (v 4.2.2), the data were analyzed. The Wilcoxon test was utilized to assess the differences between different groups. The *P* value less than 0.05 was considered statistically significant.

## Results

### Physical and Physiological Effects in the A and B Groups

**Fig. 1:**
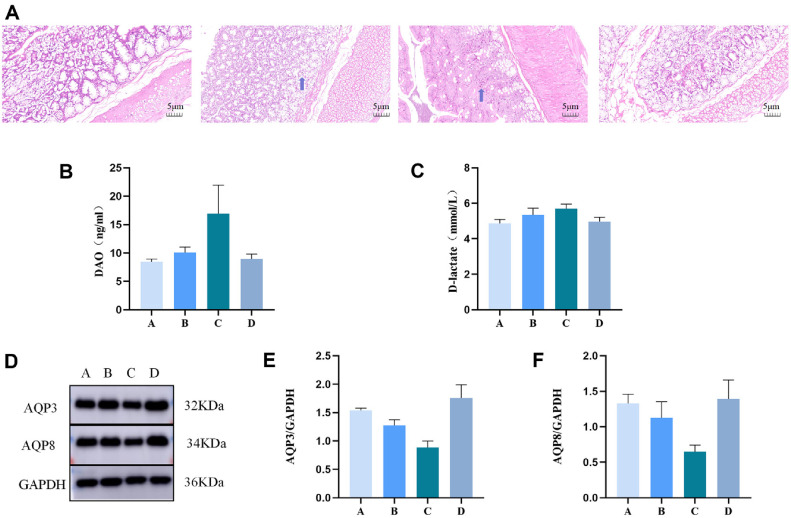
Effect of MRWD on rats with diarrhea. (**A**) Haematoxylin–eosin (**HE**) staining of colon tissue. (**B**) The concentration of DAO was detected by ELISA. (**C**) The concentration of D-lactate was detected by ELISA. (**D**) The colon samples were analyzed by western blotting using antibodies against AQP3, AQP8 and GAPDH (used as the protein loading control). (**E**) The AQP3/GAPDH ratio in the colon. (**F**) The AQP8/GAPDH ratio in the colon.

The colonic mucosal tissue of rats in group D was intact and no significant structural damage was found; the epithelial and lamina propria of rats in group C was damaged and significant inflammatory cell infiltration was seen; the mucosal tissue of rats in group B was structurally intact and a small amount of inflammatory cell infiltration was seen; and the colonic mucosal tissue of rats in group A was relatively intact and no significant inflammatory cell infiltration was seen; The levels of D-lactate and DAO, biomarkers of intestinal damage, were significantly higher in the C group than in the D group, but lower in the B and A groups (Fig. 1B and 1C). These results suggest that MRWD infusion attenuates diarrhoea-induced intestinal morphological disruption.

### Analysis of Aquaporin

Compared with D, AQP3 and AQP8 protein expression levels were significantly down-regulated in group C (*P* < 0.01); compared with group C, both group A and group B significantly up-regulated AQP3 and AQP8 protein levels (*P* < 0.05) (Fig. 1D-1F).

### Evaluation of Intestinal Microbial Diversity

The dilution curve, species accumulation curve and rank abundance curve were plotted to assess whether the sequencing sample information met the needs. The results demonstrated that the sequencing depth was appropriate, ensuring uniform richness and evenness across all samples, thereby enabling the visualization of the relationship between species diversity and sample composition (Fig. S1A-S1C).

**Fig. 2:**
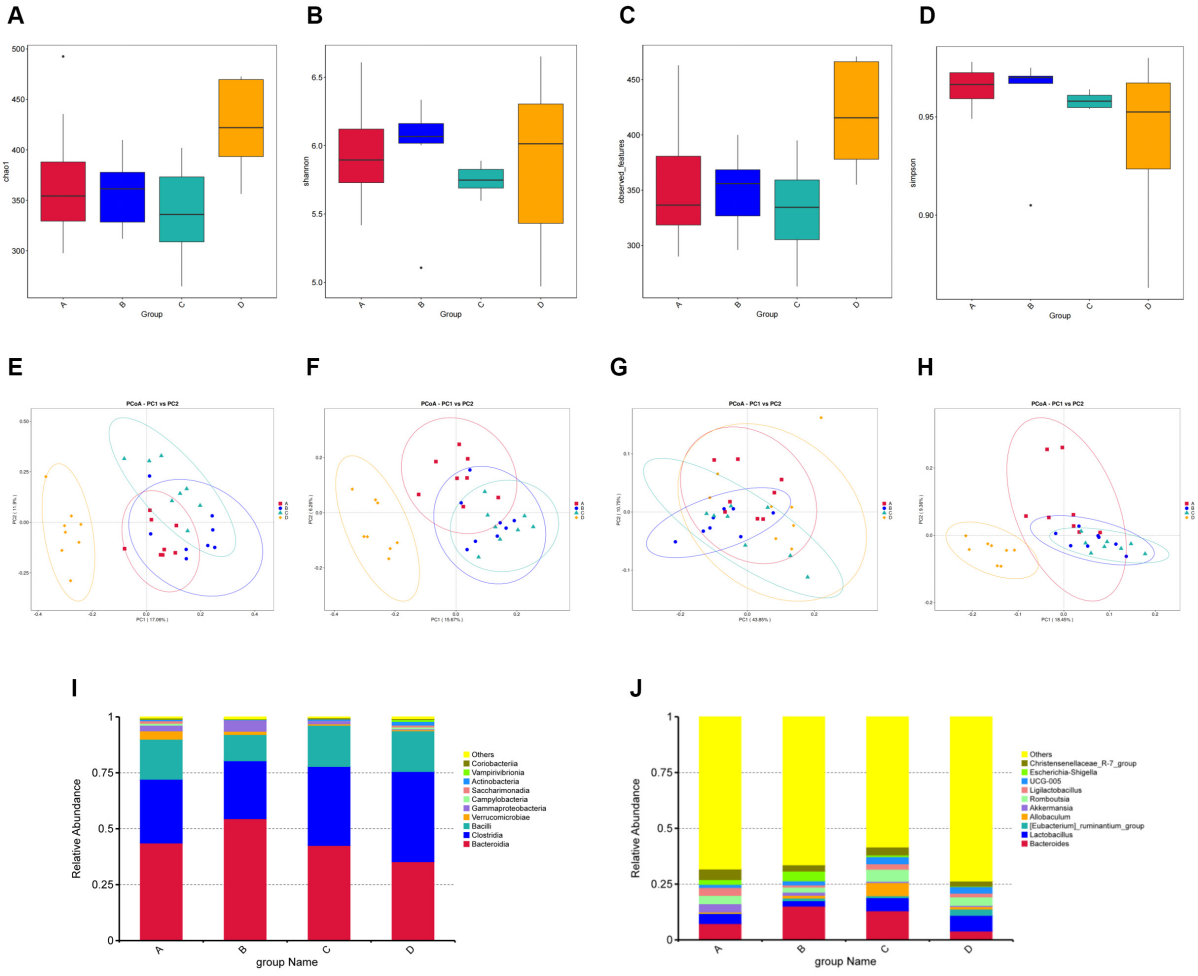
Analysis of microbial diversity and relative abundance. (**A**) Chao1 index of four groups. (**B**) Shannon index of four groups. (**C**) Observed species index of four groups. (**D**) Simpson index of four groups. (**E**) PCoA chart of the Bray-Curtis algorithm. (**F**) PCoA chart of the Jaccard algorithm. (**G**) PCoA chart of the Weighted Unifrac algorithm. (**H**) PCoA chart of the Unweighted Unifrac distances algorithm. (**I**) The relative abundance of gut microbiota at the phylum level. (**H**) The relative abundance of gut microbiota at the genus level.

Subsequently, the microbial composition diversity was assessed through Alpha and Beta diversity analyses. The results revealed that among the four indices of Alpha diversity analysis, Group A exhibited significant differences in the Chao1 index, while no significant differences were observed in Shannon, Observed species, and Simpson indices. Group B displayed significant differences in Shannon and Simpson indices but not in Chao1 and Observed species indices. No significant difference was found between Group C and Group D regarding Chao1, Shannon, Observed species, and Simpson indices (Fig. 2A-2D).

The microbial communities of the four groups exhibited significant dissimilarities across the Bray-Curtis, Jaccard, Weighted Unifrac, and Unweighted Unifrac distances in the Beta diversity analysis (Fig. 2E-2H). Moreover, all four algorithms yielded NMDS stress values ≤ 0.2, indicating robustness and reliability of the obtained results (Fig. S2A-S2D).

In addition, the relative abundance of microorganisms was further assessed. The results revealed higher microbial abundance at the phylum level in Bacteroidia, Clostridia, and Bacilli (Fig. 2I). At the genus level, Bacteroides, Lactobacillus, and Alloprevotella exhibited the highest levels of abundance (Fig. 2J).

### The Identification of Three Hub Microorganisms Was Achieved

**Fig. 3:**
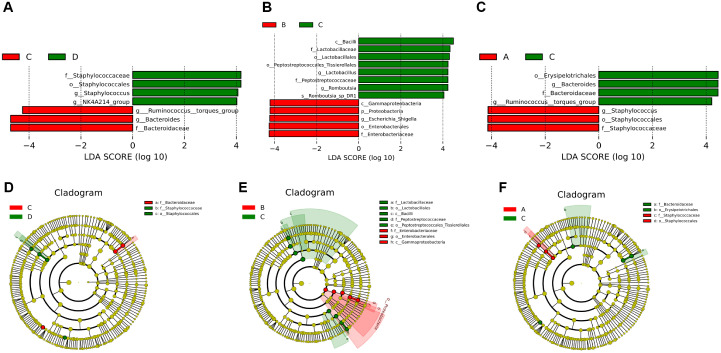
Identification of differential microorganisms between different groups. (**A-B**) Histogram of LDA value distribution and cladogram between C and D groups. (**C-D**) Histogram of LDA value distribution and cladogram between B and C groups. (**E-F**) Histogram of LDA value distribution and cladogram between A and C groups.

The LEfSe analysis, conducted at the genus level, revealed significant differences in seven DETs1 between C and D groups (Fig. 3A and 3B), 13 DETs2 between B and C groups (Fig. 3C and 3D), and seven DETs3 between A and C groups (Fig. 3E and 3F).

**Fig. 4:**
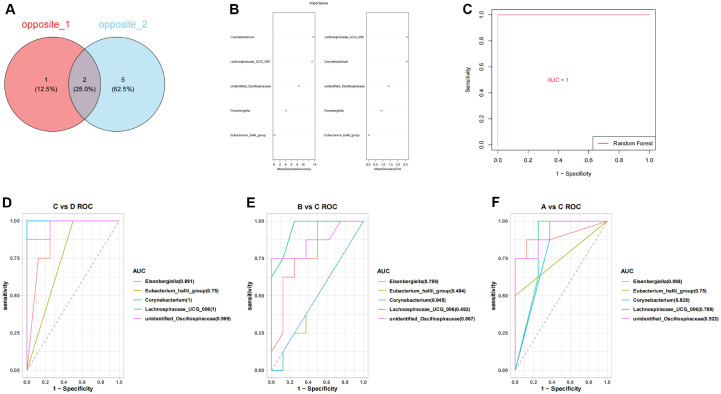
Identification of three hub microorganisms. (**A**) The Venn diagram of candidate microorganisms. (**B**) Construction of random forest (RF) model. (**C**) Receiver operating characteristic (ROC) curve of RF model. (**D**) The ability of key microorganisms to distinguish between groups C and D. (**E**) The ability of key microorganisms to distinguish between groups B and C. (**F**) The ability of key microorganisms to distinguish between groups A and C.

Subsequently, by intersecting the microorganisms exhibiting opposite expression trends between DETs1 and DETs2, we identified three opposites1: *Bifidobacterium*, *Jeotgalicoccus*, and *Aerococcus* (Fig. S3A). Similarly, through the intersection of microorganisms with contrasting expression patterns between DETs1 and DETs3, we obtained seven additional opposites2: *Eisenbergiella*, Eubacterium Hallii group, *Corynebacterium*, *Jeotgalicoccus*, Lachnospiraceae ucg-006, *Aerococcus*, and Unidentified oscillospiraceae (Fig. S3B). After that, by subtracting opposites2 and opposites1, five candidate microorganisms were obtained, including *Eisenbergiella*, *Eubacterium hallii group*, *Corynebacterium*, *Lachnospiraceae UCG-006*, and *unidentified Oscillospiraceae* (Fig. 4A). Furthermore, the RF model demonstrated that the significance of these five candidate microorganisms exceeded zero (Fig. 4B), and the AUC value in the ROC curve surpassed 0.7. Consequently, they were designated as key microorganisms (Fig. 4C). Additionally, the ability of these five key microorganisms to discriminate between groups C and D, B and C, as well as A and C was assessed. The results revealed that *Eisenbergiella*, *Corynebacterium*, and *unidentified Oscillospiraceae* exhibited AUC values exceeding 0.7 (Fig. 4D and 4F). Consequently, these three microorganisms were identified as hub microorganisms for subsequent analysis.

### Construction of OPLS-DA Model of Metabolites

**Fig. 5:**
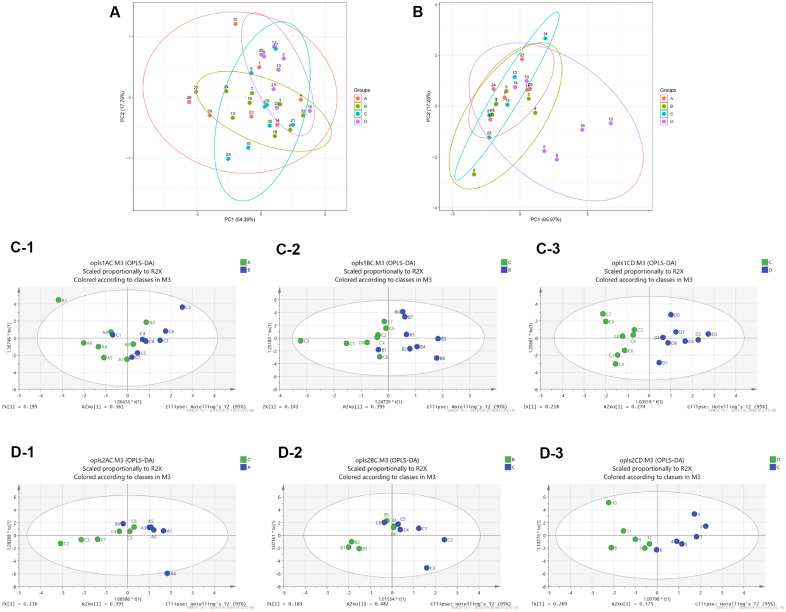
Construction of OPLS-DA model of metabolites. (**A**) Principal component analysis (PCA) of blood samples. (**B**) PCA of fecal samples. (**C**) OPLSDA maps of blood samples in different groups. (**D**) OPLS-DA maps of fecal samples in different groups.

PCA was implemented to further reflect the variability between and within the sample group as a whole. The results revealed that there were significant differences among different groups (Fig. 5A and 5B). In order to demonstrate the relationship model between metabolite expression and sample categories, as well as to effectively distinguish samples and predict their respective categories, OPLS-DA model was conducted on groups A and C, B and C, and C and D. This allowed for the determination of VIP values for each metabolite. The results revealed partial overlap but also significant differences in metabolites between blood samples from groups A and C. Moreover, notable distinctions were observed between the B and C groups, as well as the C and D groups (Fig. 5C). Significant variations were observed not only between the A and C groups but also between the B and C groups, as well as the C and D groups in fecal samples (Fig. 5D).

**Fig. 6:**
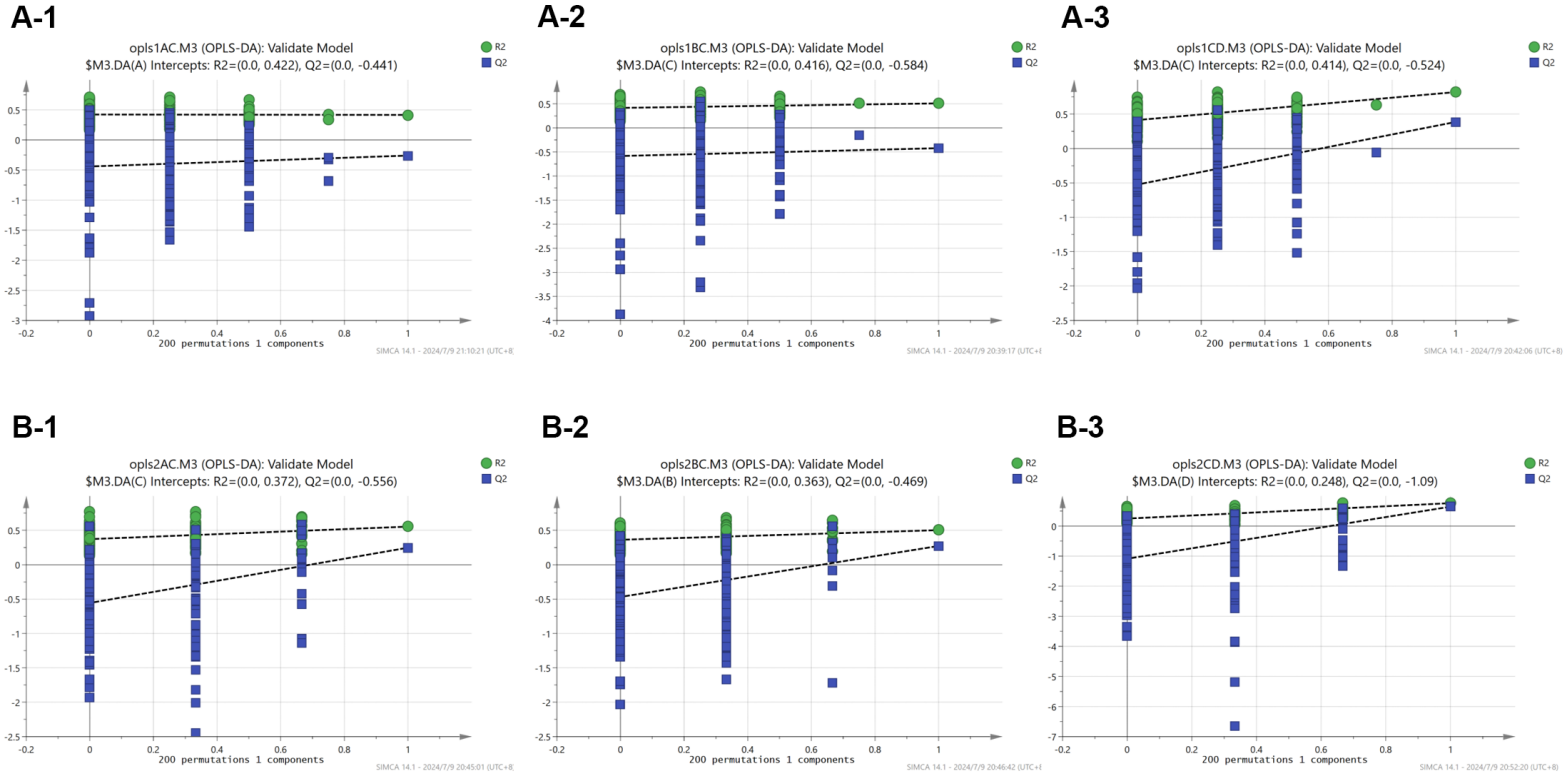
Validation of OPLS-DA model. (**A**) The permutation test of blood samples in different groups. (**B**) The permutation test of fecal samples in different groups.

The permutation test (*n* = 200) was employed to assess potential overfitting in the OPLS-DA model. The results indicated a low Q2 slope and small intercept in the blood sample, suggesting minimal overfitting (Fig. 6A). However, for fecal samples, due to limited repetitions within groups and a substantial slope intercept, the model exhibited signs of overfitting. Nevertheless, overall group differences remained significant while intra-group variations were minor (Fig. 6B).

### 2-Methylbutyrate Was Strongly associated with Hub Microbes in Blood and Fecal Samples

**Fig. 7:**
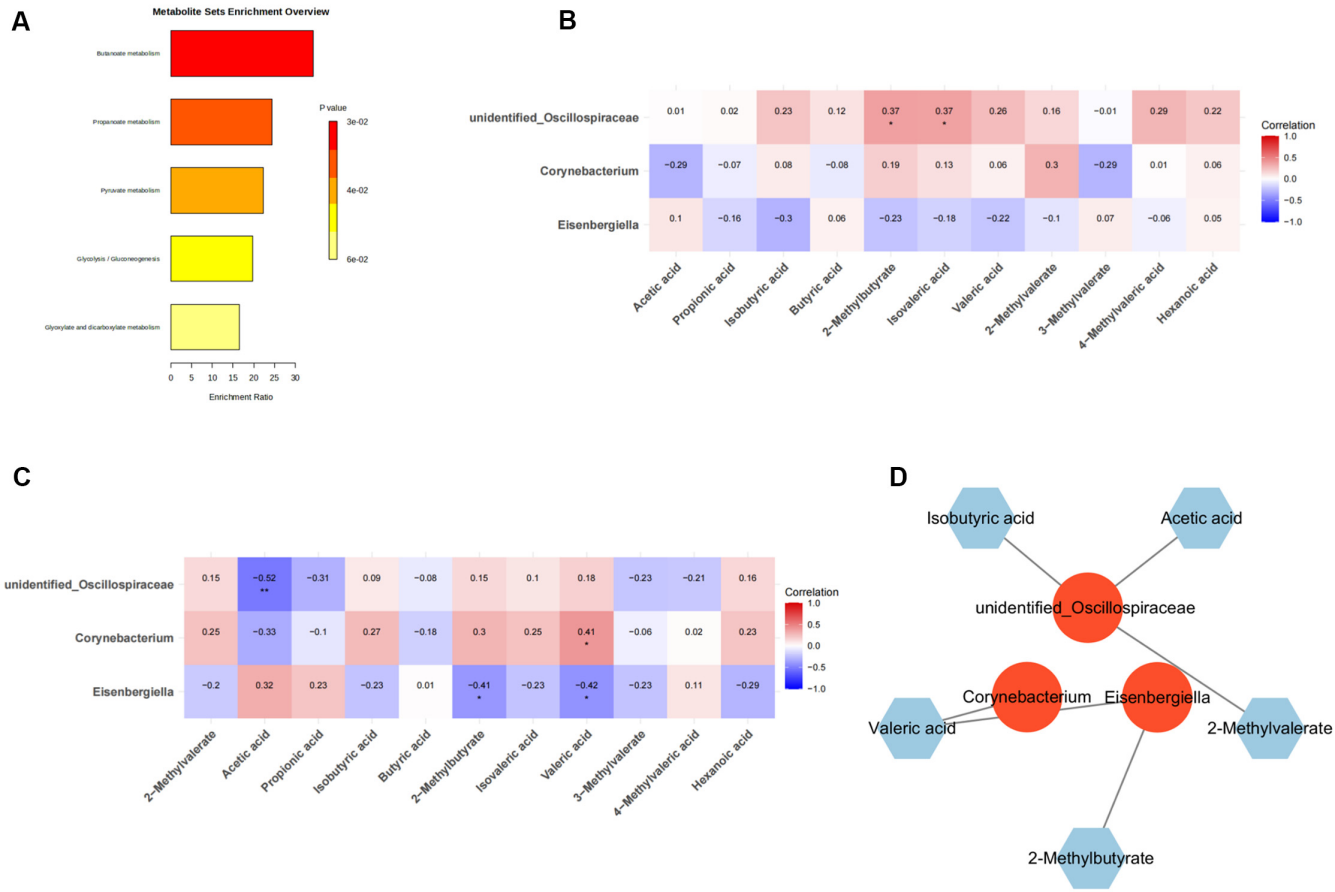
Recognition of hub metabolites. (**A**) Enrichment analysis of metabolites. (**B**) Heat map of correlation between hub microorganisms and blood metabolites. (**C**) Heat map of correlation between hub microorganisms and fecal metabolites. (**D**) The regulatory network of hub microorganisms and hub metabolites. Fig. S1 depth, sample richness and uniformity analysis of 16S gene sequencing. (**A**) The dilution curves of Chao1, Observed species, shannon and Simpson. (**B**) Species accumulation curve. (**C**) Rank abundance curve Fig. S2 nonmetric multidimensional scaling (NMDS) analysis of different groups. (**A**) NMDS chart of the Bray-Curtis algorithm. (**B**) NMDS chart of the Jccard algorithm. (**C**) NMDS chart of the Weighted Unifrac algorithm. (**D**) NMDS chart of the Unweighted Unifrac distances algorithm. Fig. S3 identification of microorganisms with opposite expression trends among different group. (**A**) The Venn diagram of opposite1. (**B**) The Venn diagram of opposite2.

Enrichment analysis indicated that metabolites were significantly correlated with metabolic pathways such as Butanoate metabolism, Propanoate metabolism, Pyruvate metabolism, Glycolysis / Gluconeogenesis, Glyoxylate and dicarboxylate metabolism (Fig. 7A). Subsequently, a strong correlation was observed between 2-methylbutyrate and Isovaleric acid with hub microorganisms in blood metabolites (Fig. 7B), while 2-methylbutyrate, acetic acid, and valeric acid exhibited a robust association with hub microorganisms in fecal metabolites (Fig. 7C). Consequently, acetic acid, isovaleric, 2-methylbutyrate, and valeric acid were designated as hub metabolites. Furthermore, hub metabolite-hub microorganism regulatory network was generated. The findings unveiled a intricate association between these hub microorganisms and hub metabolites (Fig. 7D).

## Discussion

In this study, We evaluated the role of MRWD in the treatment of diarrhea, D-LA and DAO have gained academic recognition as classical biological markers for assessing changes in intestinal permeability [14], AQPs are transmembrane proteins distributed in the intestinal mucosa that play a key role in maintaining homeostasis in the intracellular and extracellular environments of the intestinal tract [15]. Impairment of the function of AQPs can impair the mechanical barrier of the intestinal mucosa and cause a significant increase in the permeability of the intestinal mucosa [16], resulting in decreased water absorption and water accumulation in the lumen of the intestinal tract, which in turn triggers diarrhea. The present study showed that serum levels of D-LA and DAO were up-regulated in group C rats, suggesting that the intestinal mucosal permeability was elevated, while serum levels of D-LA and DAO were down-regulated in group A and B, DAO although not statistically different, was in line with the ideal tendency, suggesting that MRWD could effectively reduce the intestinal mucosal permeability and alleviate the symptoms of diarrhea, and that MRWD could reduce the symptoms of diarrhea by regulating the metabolism of the water and fluid through up-regulation of the expression of AQP3 and AQP8.We performed 16S rRNA gene sequencing based on LC-MS metabolomics approach in rats with diarrhea of qi and yin deficiency type. The results showed that there were differences in microbial composition among the four groups, and the rats in the MRWD group had reduced α and β diversity and altered gut microbiota composition, suggesting that flavouring could regulate the abundance of intestinal flora. At the phylum and genus level, rats in the flavoured ginseng and umeboshi soup group exhibited higher Bacteroidia and Bacteroides, which may have a potential effect on the production of short-chain fatty acids (SCFAs) [17], by increasing the content of SCFAs and thus reducing the inflammatory response [18], SCFAs have significant physiological and pharmacological effects, and play a vital role in maintaining intestinal health. playing a crucial role in maintaining intestinal health, and their reduction was strongly associated with the onset and progression of diarrhea. These results are consistent with previous studies [19].

The results showed that there were differences in microbial composition between the four groups. In order to find the key microorganisms among them, LEfSe analyses performed at genus level and screened showed that *Eisenbergiella*, *Corynebacterium*, and unidentified *Oscillospiraceae* in the MRWD showed a significant discriminatory ability and were identified as pivotal microorganisms. Of these, *Eisenbergiella* belongs to the phylum Actinobacteria and is thought to be involved in the production of SCFAs, and when *Eisenbergiella* is reduced, it may weaken the ability to produce SCFAs, further impairing the intestinal environment [20], and thus increasing the risk of inflammation. The relationship between bacteria in the genus *Corynebacterium* and SCFAs is a complex area, although relatively little research has been conducted on its fermentative effects, which can increase the production of SCFAs [21]. Unidentified *Oscillospiraceae*, a group of strictly anaerobic bacteria that are capable of fermenting complex plant carbohydrates in the gut, can be enhanced by increasing the production of unidentified *Oscillospiraceae* abundance, improved antioxidant capacity and SCFAs concentration, and attenuated the inflammatory response [22]. On the other hand, SCFAs have significant physiological and pharmacological effects and are considered as targets for the prevention and treatment of diarrhea, being regulated by different mechanisms in order to influence intestinal motility and enhance intestinal barrier function as well as host metabolism [23, 24]. As far as the metabolome is concerned, significant differences in metabolites were observed between the different groups. These metabolites showed significant enrichment in metabolic pathways such as butyric acid metabolism, propionic acid metabolism and pyruvic acid metabolism. Further correlation analysis showed that Isovaleric, 2-Methylbutyrate, and Valeric acid were identified as central metabolites associated with blood and faecal microorganisms, and that Isovaleric could also help to maintain the balance of the intestinal microflora by lowering the local pH and inhibiting the growth of potentially pathogenic bacteria, while promoting the proliferation of beneficial bacteria. Isovaleric can help maintain the balance of intestinal microflora and reduce diarrhea caused by dysbiosis [25, 26]. Isovaleric acid is recognized by chemosensory receptors on enterochromaffin cells, which activate neurons [27], thus maintaining intestinal health. valeric acid directly inhibits the production of pro-inflammatory cytokines (*e.g.*, IL-6), and inhibits macrophage activation by up-regulating GPR41 and GPR43 receptors [28]. This suggests that valeric acid has anti-inflammatory properties and may alleviate diarrhea symptoms by inhibiting the inflammatory response.

In the present study, we used a multi-omics sequencing approach to unearth for the first time the pivotal microorganisms and pivotal metabolites that play an important role in the intervention of MRWD in the process of diarrhea with Qi-yin deficiency, and to explore their potential roles in MRWD intervention in the process of Qi-yin deficiency syndrome with diarrhea, so that we could provide a new direction for the treatment of Qi-yin deficiency syndrome with diarrhea. Then focusing on the limitations, the limitations are: (1) Complexity: Chinese herbal medicines are complex and the mechanism of their effects on microbial abundance and diversity is not yet completely clear, and further research and validation are needed. (2) Individual differences: Due to individual differences, the therapeutic effects may vary and cannot be guaranteed to be effective for all patients. (3) Research limitations: the study on the interaction of intestinal microorganisms and metabolites is still in the preliminary stage, and has not yet fully revealed its specific mechanism of action in Qi-yin deficiency diarrhea.

## Supplemental Materials

Supplementary data for this paper are available on-line only at http://jmb.or.kr.



## Figures and Tables

**Table 1 T1:** The average body weight of rats in different treatment groups at the time of sacrifice.

Group	Average body weight (g)
A	261.88 ± 23.63*
B	260.37 ± 18.20*
C	236.50 ± 17.90##
D	360.88 ± 31.22

Values are means with standard deviations (*n* = 8).

#Compared with the D group, ^##^*p* < 0.01. *Compared with the C group, *p < 0.05.

## References

[1] Shankar S, Rosenbaum J (2020). Chronic diarrhoea in children: a practical algorithm-based approach. J. Paediatr. Child Health.

[2] Shankar S, Durairaj E (2024). Diet and management of diarrhea. Indian J. Pediatr..

[3] Tesfaw G, Siraj DS, Abdissa A, Jakobsen RR, Johansen ØH, Zangenberg M (2024). Gut microbiota patterns associated with duration of diarrhea in children under five years of age in Ethiopia. Nat. Commun..

[4] Monasterio C, Hartl C, Hasselblatt P (2020). Akute und chronische Durchfallerkrankungen: Differenzialdiagnose und Therapie [Acute and chronic diarrhea: a roadmap to differential diagnosis and therapy]. Dtsch Med. Wochenschr..

[5] Zhang WJ, Wang S, Kang CZ, Lv CG, Zhou L, Huang LQ (2020). Pharmacodynamic material basis of traditional Chinese medicine based on biomacromolecules: a review. Plant Methods.

[6] Lin TL, Lu CC, Lai WF, Wu TS, Lu JJ, Chen YM (2021). Role of gut microbiota in identification of novel TCM-derived active metabolites. Protein Cell.

[7] Ramos Meyers G, Samouda H, Bohn T (2022). Short chain fatty acid metabolism in relation to gut microbiota and genetic variability. Nutrients.

[8] Gustavsson EK, Zhang D, Reynolds RH, Garcia-Ruiz S, Ryten M (2022). ggtranscript: an R package for the visualization and interpretation of transcript isoforms using ggplot2. Bioinformatics.

[9] Mao W, Ding J, Li Y, Huang R, Wang B (2022). Inhibition of cell survival and invasion by Tanshinone IIA via FTH1: a key therapeutic target and biomarker in head and neck squamous cell carcinoma. Exp. Ther. Med..

[10] Ma R, Guan X, Teng N, Du Y, Ou S, Li X (2023). Construction of ceRNA prognostic model based on the CCR7/CCL19 chemokine axis as a biomarker in breast cancer. BMC Med. Genomics.

[11] Robin X, Turck N, Hainard A, Tiberti N, Lisacek F, Sanchez JC (2011). pROC: an open-source package for R and S+ to analyze and compare ROC curves. BMC Bioinformatics.

[12] Robles-Jimenez LE, Aranda-Aguirre E, Castelan-Ortega OA, Shettino-Bermudez BS, Ortiz-Salinas R, Miranda M (2021). Worldwide traceability of antibiotic residues from livestock in wastewater and soil: a systematic review. Animals (Basel).

[13] Shannon P, Markiel A, Ozier O, Baliga NS, Wang JT, Ramage D (2003). Cytoscape: a software environment for integrated models of biomolecular interaction networks. Genome Res..

[14] Li X, Wu Y, Xu Z, Chen J, Li Y, Xing H, Zhang X, Yuan J (2020). Effects of Hetiao Jianpi decoction on intestinal injury and repair in rats with antibiotic-associated diarrhea. Med. Sci. Monit..

[15] Kang X, Zhang H, Li X, Zhang K, Huang Z, Li Y (E2024). Electroacupuncture improving intestinal barrier function in rats with irritable bowel syndrome through regulating aquaporins. Dig Dis Sci..

[16] Kon R, Tsubota Y, Minami M, Kato S, Matsunaga Y, Kimura H (2018). CPT-11-induced delayed diarrhea develops via reduced aquaporin-3 expression in the colon. Int. J. Mol. Sci..

[17] Zhang W, Zhu B, Xu J, Liu Y, Qiu E, Li Z (2018). Bacteroides fragilis protects against antibiotic-associated diarrhea in rats by modulating intestinal defenses. Front. Immunol..

[18] Tufail MA, Schmitz RA. 2024. Exploring the probiotic potential of *Bacteroides* spp. within one health paradigm. *Probiotics Antimicrob. Proteins* **8**. doi: 10.1007/s12602-024-10370-9. Epub ahead of print. 10.1007/s12602-024-10370-9 39377977 PMC11925995

[19] Guo H, Yu L, Tian F, Zhao J, Zhang H, Chen W (2021). Effects of bacteroides-based microecologics against antibiotic-associated diarrhea in mice. Microorganisms.

[20] Li Y, Li L, Tian J, Zheng F, Liao H, Zhao Z (2022). Fiber in barley leaf attenuates hyperuricemic nephropathy by modulating gut microbiota and short-chain fatty acids. Foods.

[21] Fusco W, Lorenzo MB, Cintoni M, Porcari S, Rinninella E, Kaitsas FS (2023). Short-chain fatty-acid-producing bacteria: key components of the human gut microbiota. Nutrients.

[22] Kong Q, Chen X, Liu Y, Ali F, Idrees A, Ataya FS (2024). Sodium acetate and sodium butyrate attenuate diarrhea in yak calves by regulating gut microbiota and metabolites. Heliyon.

[23] Martin-Gallausiaux C, Marinelli L, Blottière HM, Larraufie P, Lapaque N (2020). SCFA: mechanisms and functional importance in the gut. Proc. Nutr. Soc..

[24] Facchin S, Bertin L, Bonazzi E, Lorenzon G, De Barba C, Barberio B (S2024). Short-chain fatty acids and human health: from metabolic pathways to current therapeutic implications. Life (Basel).

[25] Rondanelli M, Gasparri C, Cavioni A, Sivieri C, Barrile GC, Mansueto F (2024). A patented dietary supplement (hydroxymethyl-butyrate, carnosine, magnesium, butyrate, lactoferrin) is a promising therapeutic target for age-related sarcopenia through the regulation of gut permeability: a randomized controlled trial. Nutrients.

[26] Li X, Peng X, Qiao B, Peng M, Deng N, Yu R (2022). Gut-kidney impairment process of adenine combined with folium sennaeinduced diarrhea: association with interactions between *Lactobacillus intestinalis*, *Bacteroides acidifaciens* and acetic acid, inflammation, and kidney function. Cells.

[27] Qian JP, Jiang B, Lei XD, Tian LL, Zhou Y, Teng JQ (2023). Influence of gut microecology in the development of malignant tumors and its potential therapeutic application: a review. Medicine (Baltimore).

[28] Liu M, Zhang Y, Liu J, Xiang C, Lu Q, Lu H (2024). Revisiting the role of valeric acid in manipulating ulcerative colitis. Inflamm. Bowel. Dis..

