# Supplementary material for: Modified Renshen Wumei Decoction Improves Qi-Yin Deficiency Diarrhea by Regulating the Gut Microbiotas and Metabolites in Rats
Source: J Microbiol Biotechnol. 2025 Jun 23;35:e2412037. doi: 10.4014/jmb.2412.12037 (PMC12256838; doi:10.4014/jmb.2412.12037)
Supplement: Supplementary file 1 [file jmb-35-e2412037-supple.pdf]

J. Microbiol. Biotechnol. 2025. 35: e2412037
https://doi.org/10.4014/jmb.2412.12037

Supplementary Figures

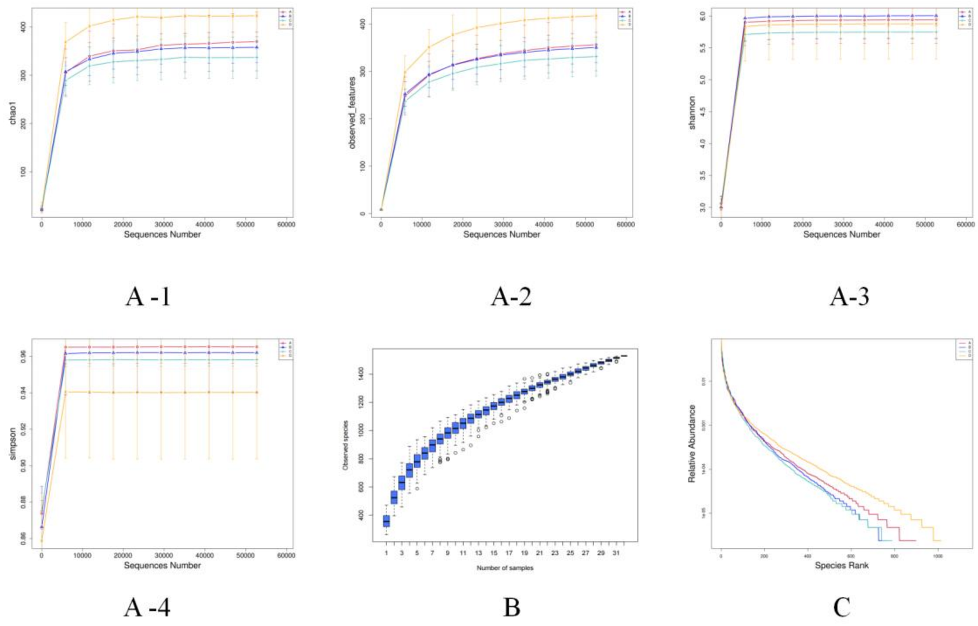

Fig. S1. Depth, sample richness and uniformity analysis of 16S gene sequencing. (A) The dilution curves of Chao1, Observed species, shannon and Simpson. **(B)** Species accumulation curve. **(C)** Rank abundance curve

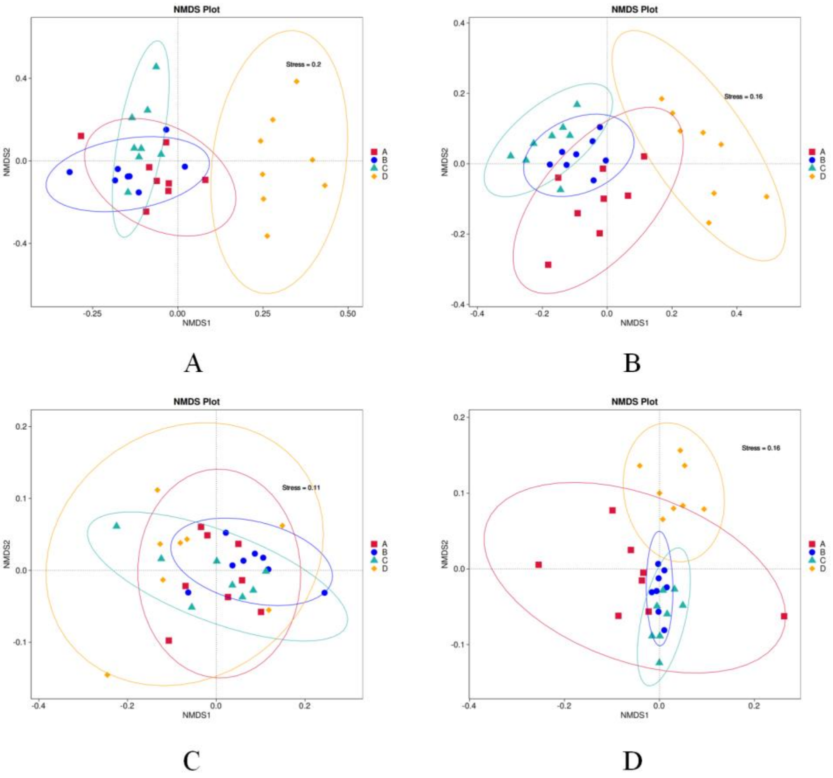

Fig. S2. Nonmetric Multidimensional Scaling (NMDS) analysis of different groups. (A) NMDS chart of the Bray-Curtis algorithm. **(B)** NMDS chart of the Jaccard algorithm. **(C)** NMDS chart of the Weighted Unifrac algorithm. **(D)** NMDS chart of the Unweighted Unifrac distances algorithm.

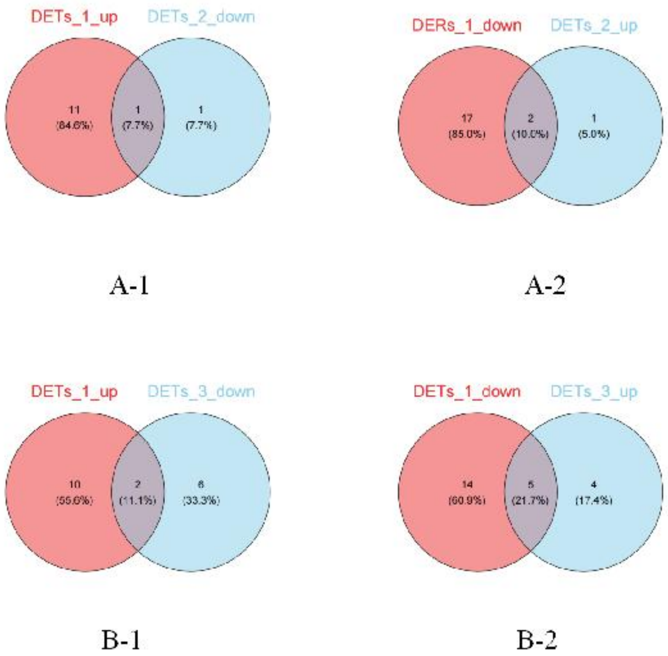

Supplementary Figure 3 Identification of microorganisms with opposite expression trends among different groups. (A) The Venn diagram of opposite1. (B) The Venn diagram of opposite2.
